# Antioxidant Compounds for the Inhibition of Enzymatic Browning by Polyphenol Oxidases in the Fruiting Body Extract of the Edible Mushroom *Hericium erinaceus*

**DOI:** 10.3390/foods9070951

**Published:** 2020-07-17

**Authors:** Seonghun Kim

**Affiliations:** 1 Jeonbuk Branch Institute, Korea Research Institute of Bioscience and Biotechnology, 181 Ipsin-gil, Jeongeup 56212, Korea; seonghun@kribb.re.kr; Tel.: +82-63-570-5113; Fax: +82-63-570-5109; 2 Department of Biosystems and Bioengineering, KRIBB School of Biotechnology, University of Science and Technology (UST), 217 Gajeong-ro, Daejeon 34113, Korea

**Keywords:** enzymatic browning, antioxidant compounds, *Hericium erinaceus*, mushroom metabolites, polyphenol oxidase, tyrosinase, laccase, natural inhibitor

## Abstract

Mushrooms are attractive resources for novel enzymes and bioactive compounds. Nevertheless, mushrooms spontaneously form brown pigments during food processing as well as extraction procedures for functional compounds. In this study, the dark browning pigment in the extract derived from the edible mushroom *Hericium erinaceus* was determined to be caused by the oxidation of endogenous polyphenol compounds by the polyphenol oxidase (PPO) enzyme family. These oxidized pigment compounds were measured quantitatively using a fluorospectrophotometer and, through chelation deactivation and heat inactivation, were confirmed to be enzymatic browning products of reactions by a metalloprotein tyrosinase in the PPO family. Furthermore, a transcript analysis of the identified putative PPO-coding genes in the different growth phases showed that tyrosinase and laccase isoenzymes were highly expressed in the mushroom fruiting body, and these could be potential PPOs involved in the enzymatic browning reaction. A metabolite profiling analysis of two different growth phases also revealed a number of potential enzymatic browning substances that were grouped into amino acids and their derivatives, phenolic compounds, and purine and pyrimidine nucleobases. In addition, these analyses also demonstrated that the mushroom contained a relatively high amount of natural antioxidant compounds that can effectively decrease the browning reaction via PPO-inhibitory mechanisms that inhibit tyrosinase and scavenge free radicals in the fruiting body. Altogether, these results contribute to an understanding of the metabolites and PPO enzymes responsible for the enzymatic browning reaction of *H. erinaceus*.

## 1. Introduction

Fungi, including yeasts, molds, and mushrooms, are attractive resources for biotechnological applications. Of these organisms, many mushroom species have historically been used for food or medicinal purposes. Approximately 140,000 mushroom species belonging to the phyla Ascomycetes and Basidomycetes have been identified [1]. Over 2000 mushroom species are edible, and at least 10% of these species have been used traditionally for food or medicine [1]. Moreover, mushrooms produce small bioactive molecules such as secondary metabolites and polysaccharides that can be used for their medicinal properties in antitumor, immunomodulatory, antibacterial, and antiviral treatments [1,2,3,4].

*Hericium erinaceus* (also called lion’s mane mushroom, bearded tooth mushroom, satyr’s beard, bearded hedgehog mushroom, pom pom mushroom, or bearded tooth fungus) is an edible and medicinal mushroom found in East Asia (China, Korea, and Japan) that belongs to the tooth fungus group [5]. This mushroom species has a non-typical mushroom shape, with no cap and no stem. It is found on hardwoods and identifiable as a single clump within long dangling spines. The mushroom fruiting body is mainly white, although it becomes brown or yellow with age. In East Asia, *Hericium* species mushrooms are highly valued for their medicinal properties and have been used in traditional Chinese medicine [4]. In the past few decades, there has been considerable research interest in the bioactive properties of this species [5]. Several compounds isolated from *H. erinaceus* have been found to have biological activities, including antitumor, antimicrobial, antioxidant, and cytotoxic activities [5,6,7,8].

The mushroom fruiting body is usually white initially, but, when processed for food, the mushroom sometimes develops brown to black pigments. Indeed, in the initial purification process for both proteins and bioactive compounds, the solution extracted from the mushroom lysate is pale yellow, and the extract darkens over time during these processes [9]. The oxidation reaction of polyphenols and other oxidizable substances can potentially be prevented by antioxidant compounds in the mushroom fruiting body. However, oxidizable and aromatic-ring-containing substances in the mushroom fruiting-body extract can be exposed, resulting in their oxidization under aerobic conditions. Similarly to well-known plant substances [10], a number of aromatic compounds, including hericines, erinacines, alkalonides, lactones, and their derivatives [5], can presumably be converted to pigment-forming molecules. These mushroom metabolite substances are possibly oxidized products that are formed by polyphenol oxidases (PPOs) in the mushroom fruiting body. However, there are no reports on these pigment-forming compounds or the potential enzymes in *H. erinaceus*.

PPOs, including tyrosinase and laccase, are enzymes well-known for their activities related to post-harvest browning pigments in agricultural products [11]. Although these pigment compounds, such as certain secondary metabolites, might be useful for enhancing bioactive compounds in fermentation processes or for helping to preserve protein in forage crops, the browning reactions are generally viewed negatively in food processing or in fresh products [10,11,12]. The dark color reaction occurs via PPOs, which can be induced by wounding or pathogen attacks as part of the defense response in plants and fungi [10,12]. Indeed, it has been shown that PPOs play such a role in the white button mushroom (*Agaricus bisporus*) [13]. Nonetheless, the exact kinds of substances in the mushroom that are converted to brown products by some PPOs are unknown in mushroom species.

In our previous study, products of the dark browning reaction were observed as interfering substances during the purification process for a lectin protein from the fruiting-body lysate of *H. erinaceus* [9]; the chromatography fractions were dark brown with a gradient in the upper part. However, there is a lack of information available about this coloration event in the mushroom fruiting-body extract. Therefore, the aim of this study was to identify the cause of the dark brown pigmentation via the endogenous enzymatic reaction of the PPO family with oxidation activity, and to reduce the occurrence of this pigmentation. Furthermore, the putative PPO-coding genes and their transcripts in both the fruiting body and the mycelium were analyzed as putative proteins involved in the enzymatic browning reaction in the mushroom. In addition, a mushroom metabolite analysis was performed to identify natural compound families in the fruiting body and mycelium of *H. erinaceus* as potential antioxidant substances, or as the browning-reaction substrates of the PPOs.

## 2. Results and Discussion

### 2.1. Observation of Dark Brown Pigment in the Fruiting-Body Lysate

**Figure 1:**
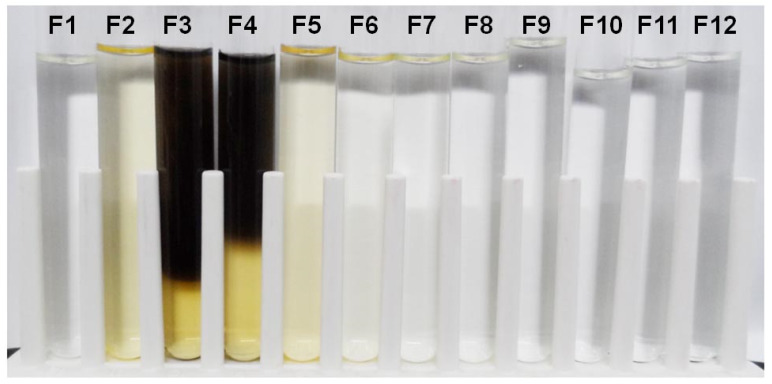
The fruiting body extract fractions (F1–F12) eluted from an ion-exchange column, DEAE-Sepharose, in the edible mushroom *Hericium erinaceus*, with dark brown pigments in F3 and F4.

The fruiting body of the edible mushroom *H. erinaceus* is normally white. Its color becomes yellow or brown over time. In the preparation of the fruiting-body lysate for protein purification, the color of the mushroom crude lysate was initially pale yellow. However, over time, the color of the DEAE-sepharose column flow-through fractions collected during protein purification became dark brown (Figure 1). The dark brown color was observed in Fractions 3 (F3) and 4 (F4). Interestingly, the color pigments localized to the upper part of the test tubes, where the surface was exposed to air, which created a gradient from top to bottom. The yellow color remained at the bottom of the F3 and F4 tubes, but the dark pigmentation progressed over time. This dark brown pigmentation pattern implied that the pigmentation process involves oxygen and certain enzymes or proteins found in F3 and F4.

### 2.2. Measurement of Dark Brown Pigment

To measure the relative amount of the brown pigment, the absorbance of the fractions was scanned by ultraviolet-visible (UV-vis) spectroscopy in the wavelength range of 210–750 nm. However, the detection wavelength for the pigment was less than 210 nm (data not shown). Therefore, UV-vis spectrophotometry was not useful for measuring the pigmentation, because a mixture of proteins as well as pigment products were present in the fraction (data not shown). The proteins were interfering substances to detect the pigment in the mushroom extract at 210 nm. 

**Figure 2:**
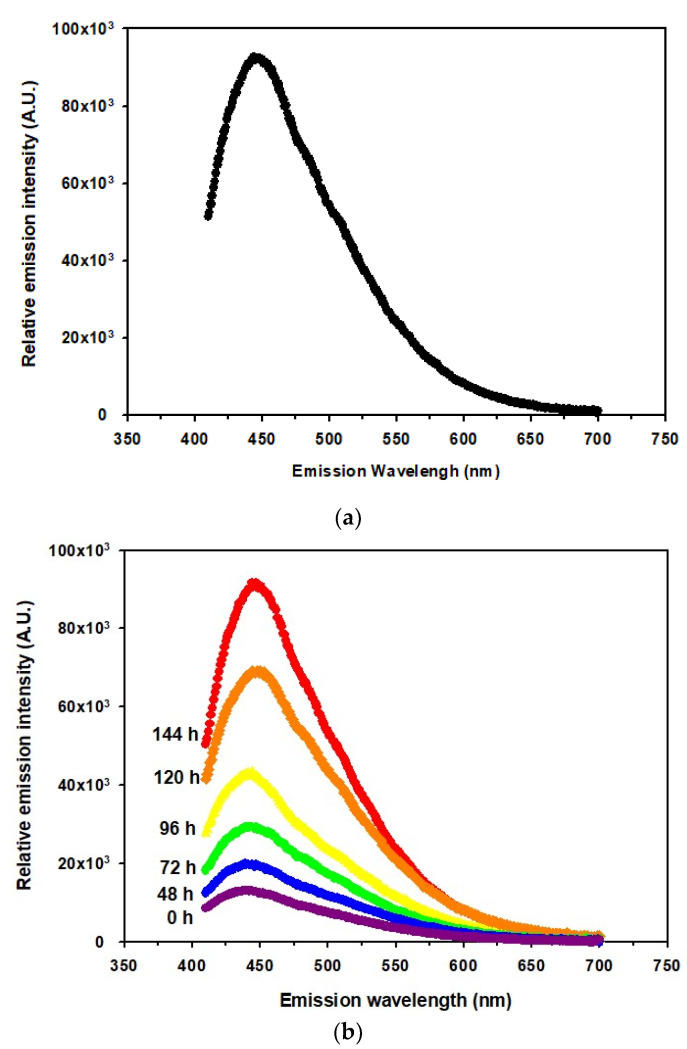
Fluorescence emission spectra of the dark brown pigment in the protein fraction. (**a**) Emission spectrum of the dark pigment fraction scanned by a fluorospectrophotometer with an excitation wavelength of 380 nm. (**b**) Fluorescence spectra of the pigment fractions for relative quantification of the brown pigment, which increased in a time-dependent manner under the same conditions. The fluorescence intensity of every fraction was measured with the following time points: initial 0 h, purple; 48 h, blue; 72 h, green; 96 h, yellow; 120 h, orange; 144 h, red.

An alternative method, fluorescence spectroscopy, was used to detect the brown pigmentation. The fractions with dark brown pigment were scanned. To determine the optimum emission and excitation wavelength for the pigment, the emission wavelength (*Em*) was fixed at 460 nm and the excitation (*Ex*) spectra ranged from 250 to 420 nm was scanned. The highest fluorescence was obtained at 380 nm as the optimal emission wavelength. The excitation wavelength was then fixed at 380 nm, and the emission spectra were scanned from 420 to 700 nm. The maximum intensity signal was detected at the emission wavelength at 444 nm (Figure 2a) setting the excitation wavelength at 380 nm. The emission intensity of each sample corresponded to the relative amount of brown pigmentation, which formed in a time-dependent manner (Figure 2b).

**Figure 3:**
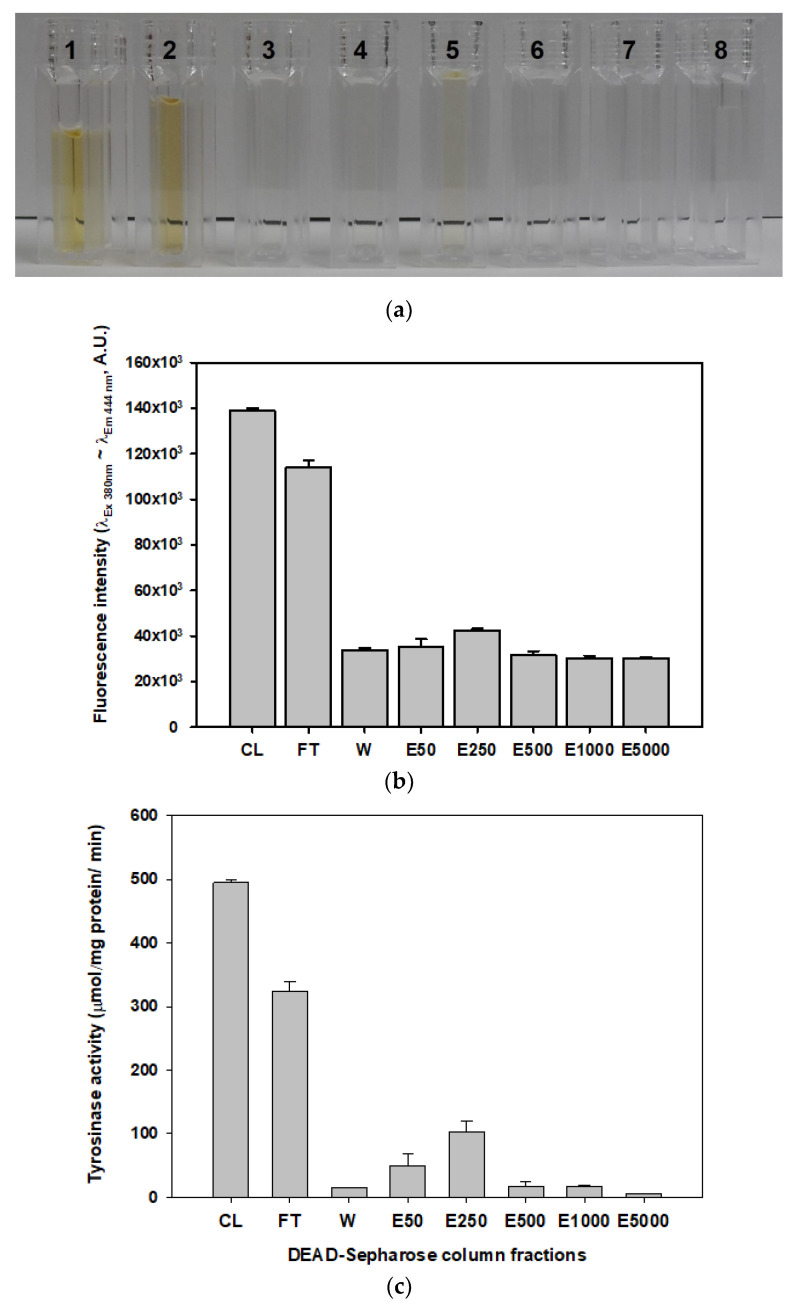
Measurement of the browning pigment substances and tyrosinase activities in the fraction pools collected in the DEAE-Sepharose column. (**a**) The protein fraction pools eluted from the DEAE-Sepharose column: 1, crude extract fraction pool (CL); 2, flow-through fraction pool (FT); 2, wash fraction pool (W); 4–8, elution fraction pools with 50 mM (E50), 250 mM (E250), 500 mM (E500), 1000 mM (E1000), and 5000 mM NaCl (E5000), respectively, in 50 mM Tris-HCl buffer. (**b**) Fluorescence intensities (Arbitrary Unit, A.U.) of these fraction pools for the measurement of brown pigment under the optimum conditions with λ_Ex380 nm_~λ_Em460nm_. (**c**) Tyrosinase-specific activities of these fraction pools under the enzyme assay conditions described in the material and method section.

The fluorescence intensity of fraction pools collected was measured at *Ex*_380nm_~*Em*_444nm_. All fractions exhibited positive fluorescence intensity readings. The visible intensity of the dark brown pigmentation seemed to correlate with the measured fluorescence intensity for all fractions (Figure 3a). The maximum fluorescence intensity was observed in the pooled crude (CL) fraction (Figure 3b). The fluorescence intensity in the CL fraction was 1.4-fold higher than that in the pooled flow-through (FT) fraction; the CL fraction showed higher fluorescence intensity than the other fractions eluted from the DEAE-sepharose column. The fluorescence intensities of both the CL and FT fractions were 2.7–3.6 times higher than those in the NaCl elution fractions. Although the elution fraction (E250) with 250 mM NaCl was a light yellow color (Figure 3a), the fluorescence intensity of this fraction was lower than that of the CL or FT fractions. However, the E250 fraction showed an approximately 1.2–1.3-fold higher intensity than the other NaCl elution fractions (E50, E500, E1000, and E5000). All of the NaCl elution fractions, except for the E250 fraction, appeared clear, but a fluorescence signal from the dark pigmented compounds was still detected.

The fluorescence spectra of the dark brown pigment suggested that the compounds responsible for the pigmentation might be aromatic or heterocyclic aromatic compounds [14]. Mushrooms contain a considerable number of phenolic compounds, such as flavonoids [5,7,8]. These compounds are antioxidant substances in mushrooms, but are also good substrates for polyphenol oxidases (PPOs) [15]. The mechanisms by which the dark pigment is formed in *H. erinaceus* are not clearly understood, but browning in cultivated button mushrooms (*Agaricus bisporus*), an extensively cultivated mushroom, is induced by bacterial infection and oxidation after harvesting [16]. It has reported that the brown pigments form as a defense mechanism when mushroom tissue becomes damaged or infected [16]. This pigmentation is mainly caused by the oxidation of endogenous phenolic substances, which are enzymatically catalyzed by PPOs, including tyrosinases and laccases. 

### 2.3. Measurement of Tyrosinase Activity and its Deactivation in the Mushroom Extract

To test whether PPOs were responsible for the pigmentation occurring in *H. erinaceus,* the enzymatic activity of the tyrosinase belonging to the PPO family was measured in the fractions eluted from the DEAE-sepharose column (Figure 3c). The CL fraction showed the highest tyrosinase activity per unit protein in all of the fractions tested. In addition, the FT fraction had higher enzyme activity than other fractions. However, tyrosinase activities in the NaCl elution fractions were much lower than those in the CL or FT fractions. The tyrosinase activity values correlated with the fluorescence intensity values in each fraction described above (Figure 3b). These results could suggest that the dark brown pigmentation that occurs in *H. erinaceus* could be due to enzymatic reactions catalyzed by tyrosinases. It seems the CL and FT fractions could contains tyrosinases with natural substrates such as phenolic compounds to convert the brown pigment. However, the NaCl eluted fractions contained low amount of the enzyme as well as less amount of the low molecule substances, which were already pass through the column. Therefore, the intensity of the brown pigment in the elution fractions would lower than the CL and FT fractions.

**Figure 4:**
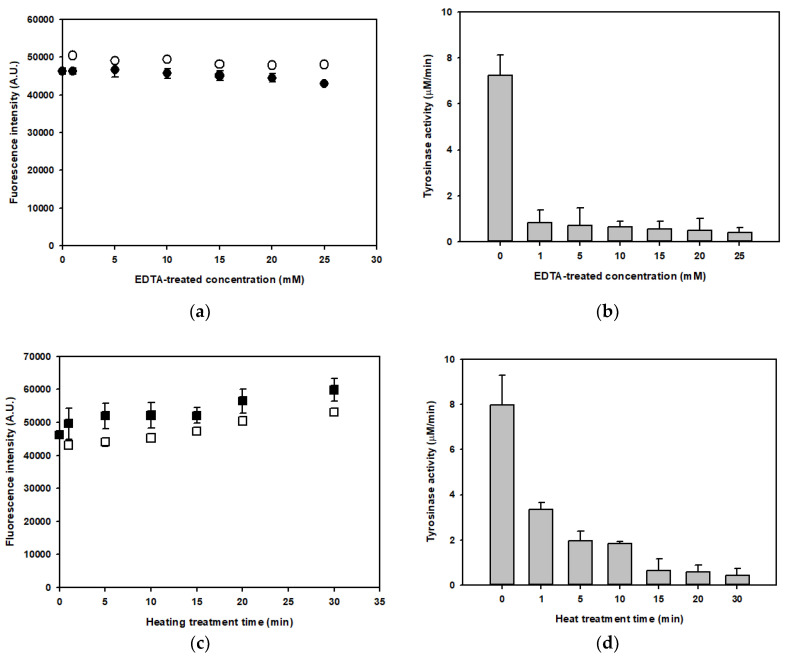
Measurement of fluorescence intensities and tyrosinase activities in the mushroom lysate treated with EDTA or heat. (**a**) Fluorescence intensities of the EDTA-treated lysates at the initial time (black circles) and 14 days later (white circles). (**b**) Total tyrosinase activities of EDTA-treated lysates. (**c**) Fluorescence intensities of the heat-treated lysates at the initial time (black squares) and 14 days later (white squares). (**d**) Total enzyme activities of the heat-treated lysates. Fluorescence intensities (Arbitrary Unit, A.U.) and tyrosinase activities of all fractions were measured under the optimum conditions with λE × 380 nm~λEm 460 nm and the enzyme assay conditions described, respectively.

In addition, to confirm that the dark brown pigment was an enzymatic reaction product, the endogenous tyrosinase in the mushroom lysate was immediately deactivated or inactivated. The chelating agent ethylenediaminetetraacetic acid (EDTA) can eliminate metal ions in a copper-containing metalloprotein to deactivate tyrosinase [15,16]. EDTA was added in concentrations ranging from 1 to 25 mM into the mushroom lysate. At the initial time-point, the fluorescence intensities in EDTA-treated samples (black circles) and untreated samples were slightly different (Figure 4a). There was a slight trend towards decreasing fluorescence intensity, depending on the concentration of EDTA. After 14 days, the fluorescence intensity (white circles) did not increase significantly (less than 10%). With the chelation agent added to the lysate, tyrosinase activity was strongly inhibited (Figure 4b). Even low concentrations (1 mM) of EDTA abolished the tyrosinase activity by up to 90%.

For the inactivation of tyrosinase activity, the mushroom lysate was treated at 100 °C for 1 to 30 min. The fluorescence intensity and the tyrosinase activity in the heat-treated lysates were monitored. The fluorescence intensity (black squares) gradually increased in the heat-treated samples, depending on the incubation time (Figure 4c). The fluorescence intensity of the lysate heated for 30 min increased 1.3-fold compared with that of non-heated samples. The heat treatment may have acted to enhance the browning pigment responsible for the fluorescence intensity in these samples. Heat treatment reportedly changed the composition of polyphenolic compounds in shiitake mushroom extracts [17]. The polyphenolic content of shiitake extracts was enhanced with heating temperature and treatment time. However, after 14 days, the fluorescence intensity (white squares) of the heat-treated lysates had decreased by less than 10–13%. Moreover, heat treatment was effective with respect to tyrosinase activity, which decreased by 58.0% after 1 min, 75.2% after 5 min, and 76.8% after 10 min at 100 °C (Figure 4d). The enzymes in the samples treated for 30 min at 100 °C were almost completely deactivated, and the residual enzyme activity was 5.5%. However, the enzyme activity in the heat-treated samples was higher than that in EDTA-treated samples. The heat treatment did not completely inactivate the enzyme. In addition, the heat treatment could have influence on increasing spontaneously brown coloring by the chemical reactions such as sugar-amine reactions in the endogenous substance of the mushroom extract. These results indicate that the brown coloring pigmentation in *H. erinaceus* extracts could be the product of an enzymatic reaction by a tyrosinase belonging to the PPO family of enzymes.

### 2.4. Analysis of the Transcription Levels of PPO-Coding Genes in the Different Growth Phases of the Mushroom

**Figure 5:**
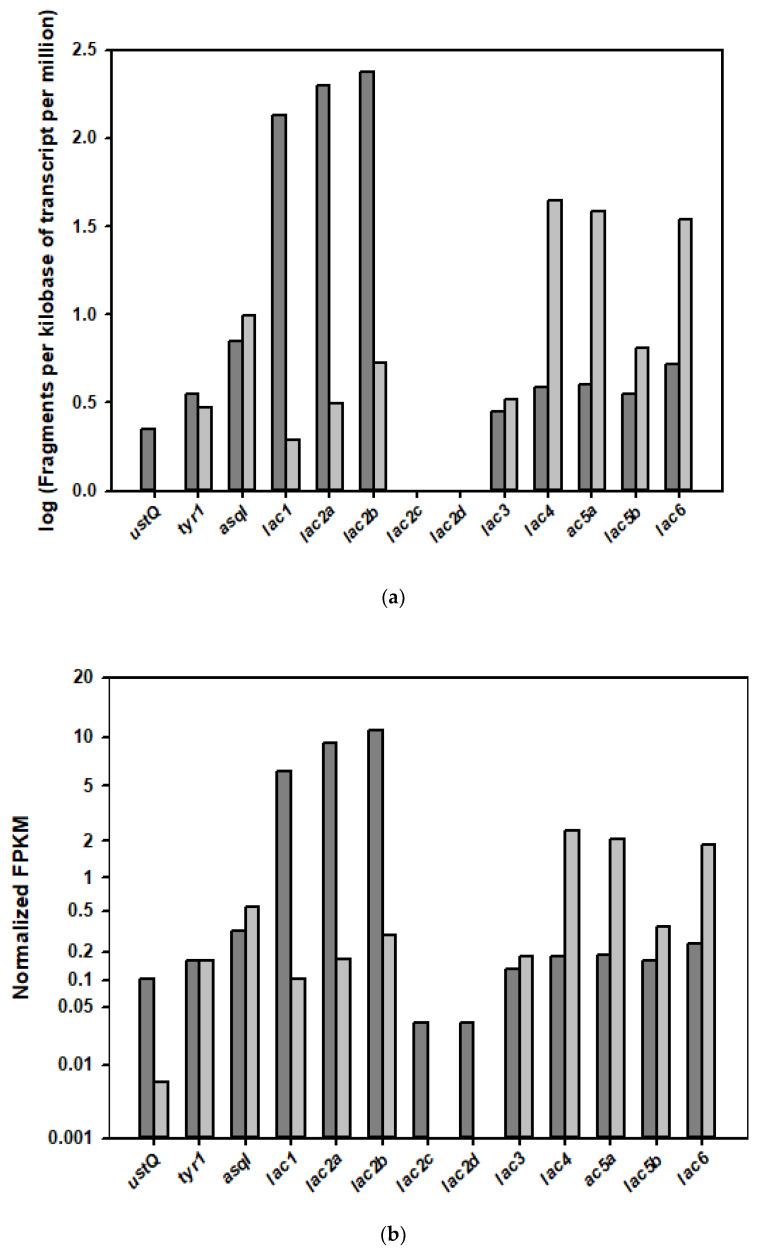
Transcription levels of tyrosinase- and laccase-coding genes involved in the enzymatic browning reaction in the fruiting body (dark gray) and the mycelium *(gray-white) of H. erinaceus*. (**a**) Log scale for transcription levels expressed as fragments per kilobase of transcript per million (FPKM). The log (FPKM) values of *lac2c* and *lac2d* were less than −0.1. (**b**) Normalized FPKM values relative to a suite of endogenous actin genes in each growth phase.

To identify the correlation of the PPO-coding cDNAs with the dark browning event, the expression levels of the tyrosinase and the laccase genes in the RNA-seq data of the fruiting body and the mycelia were analyzed (Figure 5). The isotranscript genes for tyrosinase and laccase were differentially expressed in the growth phases; among seven putative tyrosinase-coding genes identified from expressed transcripts in the mushroom genome, three of them, *ust*Q, *tyr*1, and *asq*I, were observed in both the fruiting body and mycelium. Interestingly, the expression of *ust*Q was approximately 20-fold higher in the fruiting body than in the mycelium, whereas the expression of the *tyr*1 gene was about 1.1-fold higher. However, the expression of another tyrosinase gene (*asq*I) was 1.4-fold lower in the fruiting body than in the mycelium. The *ust*Q-encoded tyrosinase could be a major PPO enzyme involved in the enzymatic browning reaction in the *H. erinaceus* fruiting body.

Thirteen putative laccase-coding genes were identified in the genome, but only 10 transcripts were expressed in the fruiting body and mycelium. These transcripts were differentially regulated in different growth phases (Figure 5). In the fruiting body, five laccase transcripts were upregulated, and four other genes were downregulated. Interestingly, the *lac1*, *lac2a*, and *lac2b* transcripts had significantly higher isotranscript expression in the fruiting body; the transcription levels of *lac1*, *lac2a*, and *lac2b* genes were upregulated 59-, 55-, and 38-fold in the fruiting body compared with the mycelium. Among these more highly expressed transcripts, *lac2* genes were expressed as four isotranscripts (*lac2a, lac3b, lac2c*, and *lac2d*), and the expression levels of all of these transcripts were higher in the fruiting body. On the other hand, five other laccase-coding transcript isoform genes were downregulated in the fruiting body, while these transcripts were highly expressed in the mycelium. Two transcripts, *lac4* and *lac5a*, among the five genes that were upregulated in the mycelium, had about 10-fold higher expression in the mycelium growth stage. The transcripts of *lac3*, *lac5b*, and *lac6* had 1.4-, 2.1-, and 7.7-fold higher expression in the mycelium. 

These transcriptional analyses showed that the laccase transcripts were also highly expressed and regulated in the growth stages. Moreover, the laccase isoenzymes translated from the upregulated transcripts could be potential PPOs involved in the enzymatic browning reaction in the fruiting body. 

### 2.5. Analysis of the Main Metabolites in the Mushroom Fruiting Body and Mycelium

To analyze the potential substances involved in the enzymatic browning reaction or compounds with protective roles in the mushroom fruiting body and mycelium, a metabolomic analysis was performed in two different growth phases using CE-TOF-MS and LC-TOF in two modes for cationic and anionic metabolites with molecular weights ranging from 50 to 1000 *m/z*. In the fruiting body, CE-TOF-MS detected 198 metabolites (138 metabolites in cation mode and 60 metabolites in anion mode), and LC-TOF-MS identified 63 metabolites (26 metabolites in positive mode and 37 metabolites in negative mode). On the other hand, in the mycelium, CE-TOF-MS detected 171 metabolites (107 metabolites in cation mode and 64 metabolites in anion mode), and LC-TOF-MS revealed 38 metabolites (12 metabolites in positive mode and 26 metabolites in negative mode). 

Among these metabolites, well-known natural compounds with tyrosinase-inhibitory activity and antioxidant activity (Table 1) and low-molecular-weight substances potentially involved in the enzymatic browning reaction (Table 2) were identified, respectively, in the mushroom fruiting body and the mycelium. Interestingly, the antioxidant and enzymatic browning substances presented in a growth-stage-dependent manner. For protection against the endogenous browning reaction in the mushroom, 18 putative antioxidants substances were detected and categorized into tyrosinase inhibitors, antioxidants/free-radical scavengers, and polyamines (Table 1). A large number of metabolites with antioxidant activity were found in the fruiting body compared with in the mycelium (17 vs. 9), with an overlap of eight compounds. Of these antioxidant substances, ascorbic acid, caffeic acid, chlorogenic acid, curcumin, apyridoxal, and quinic acid—natural compounds with tyrosinase-inhibitor activity—were detected in the mushroom fruiting body, while vallinic acid was only found in the mycelium. However, terephalic acid was detected in both mushroom development stages. As tyrosinase inhibitors, the levels of ascorbic acid and quinic acid were significantly higher than those of other compounds in the fruiting body.

Moreover, adenosine, ergosterol, erothioneine, glutathione, and 5-hydroxytryptophan were commonly observed as other antioxidants/free-radical scavengers in both the fruiting body and the mycelium (Table 1). In terms of the differences among these compounds, the amount of erothioneine was 29 times higher, whereas the content of glutathione was 20 times lower in the fruiting body than in the mycelium. These data may indicate that different compounds in different mushroom growth stages are synthesized as antioxidant substances for the inhibition of the enzymatic browning reaction or for the scavenging of free radicals. 

On the other hand, for the enzymatic browning products, free amino acids and their derivatives, phenolic compounds, and purine and pyrimidine nucleobases were identified as potential substrates (Table 2); these compounds were less abundant in the fruiting body than in the mycelium (18 vs. 29), with an overlap of 12 compounds. Interestingly, the compounds that were grouped as free amino acids and their derivatives were predominantly found in the fruiting body, while phenolic compounds and purine/pyrimidine nucleobases were mainly detected in the mycelium. Phenylalanine and tyrosine were the main substances in terms of the relative amounts of these compounds. These amino acids could be used as substrates for the enzymatic browning reaction by PPOs, including tyrosinase and laccase. Both phenylalanine and tyrosine are favorable substrates and initial precursors for the dark pigment in the melanin synthesis pathway [18,19,20]. In addition, purine and pyrimidine compounds that contain a pentose sugar—ribose or deoxyribose—with an amine group in their backbone structures are also known to be potential substrates for browning reactions via the Maillard reaction under high temperatures [21]. Adenosine nucleoside phosphate derivatives—AMP, ADP, and ATP—were the main compounds in the nucleobase family. They could be putative browning substances, since their levels were approximately 10 times higher in the fruiting body than in the mycelium. These metabolite analysis results showed that the fruiting body and the mycelium can biosynthesize endogenous antioxidant compounds to reduce the browning reaction, whereas synthesized metabolites such as aromatic amino acids, nucleobases, and their derivatives might be prospective substrates of PPOs for the enzymatic browning reaction.

In this study, to identify the browning reaction in the mushroom extract through the endogenous enzymatic reaction of the PPO-family enzymes with oxidation, a method was established to quantitatively measure dark brown pigmentation in mushroom fruiting-body lysate using a fluorospectrophotometer. Excitation and emission wavelengths of 380 nm and 444 nm, respectively, were found to be the most efficient for measuring this pigmentation. Compounds that fluoresce between these wavelengths generally have aromatic functional groups, aliphatic or alicyclic carbonyl structures, or highly conjugated double-bond structures with low energy-transition levels [22]. Although both the pH and solvent affected the fluorescence readings in these samples, the wavelengths used in this study quantitatively detected the dark brown pigments. Interestingly, the wavelengths used (λ_Ex380nm_ ~ λ_Em460nm_) were close to those used to detect cyclized *o*-quinone oxidation products (aminochrome, dopachrome, and furanoquinone), generated from the tyrosinase-catalyzed oxidation of dopamine, L-DOPA, and 3,4-dihydroxyphenylacetic acid [23]. These cyclized *o*-quinone oxidation products are eventually polymerized to melanin [23].

The pigment products were estimated to be oxidized polyphenol compounds similar to melanin, derived from PPOs in the mushroom lysate [24]. PPOs catalyze enzymatic browning by oxidizing phenolic compounds to their respective *o*-quinones, which subsequently undergo nonenzymatic oxidation to brown melanin pigments or participate in addition–polymerization reactions with protein functional groups to form cross-linked polymers [25]. This phenomenon is a major problem in the processing of both fruits and vegetables [26]. Moreover, both tyrosinase and laccase are well-known oxidase enzymes that cause brown pigmentation in mushroom species [15,16]. 

Tyrosinases (monophenol, *o*-diphenol:oxygen oxidoreductase, EC 1.14.18.1) are type-3 copper proteins that are involved in the initial step of melanin synthesis [15]. These enzymes catalyze both the *o*-hydroxylation of monophenols and the subsequent oxidation of the resulting *o*-diphenols into reactive *o*-quinones, which evolve spontaneously to produce intermediates that are associated with dark brown pigments [15]. In mushrooms, tyrosinases are endogenous biological factors that are associated with the formation and stability of spores in defense and virulence mechanisms, and in browning and pigmentations [27,28,29]. Although the catalytic mechanisms of these enzymes are still unknown, the deactivation and inactivation of the metalloprotein tyrosinases in the mushroom lysate seen in this study may be a way to prevent browning, because neither the EDTA-treated nor heat-treated lysate became pigmented. 

The other enzyme type, laccase (EC 1.10.3.2), is a multicopper oxidase that is capable of oxidizing a wide range of aromatic compounds [30]. These enzymes also catalyze the oxidation of *o*- and *p*-diphenols into their corresponding quinones for the enzymatic browning reaction. Although the roles of plant laccase in the radical coupling of monolignols to form lignin and flavonoid polymerization in the cell wall [31] and in the protection of plants against environmental stresses [32,33] are well-understood, the in vivo biological functions of mushroom enzymes belonging to the PPO family are still unclear. Nevertheless, the broad substrate specificities of laccases make them enzymes with potential involvement in the pigment formation via oxidation of endogenous aromatic compounds in mushrooms [34].

According to the expression pattern in the fruiting body and mycelium growth phases, a transcription analysis was performed using RNA-seq sequencing, which showed that the number of laccase transcripts was more diverse than tyrosinase transcripts, and the expression levels of certain laccase genes, namely lac1, lac2a, and lac2b, were significantly increased in the fruiting body compared with levels in the mycelium (Figure 5). As PPOs, these laccases would be abundant in the tissue of the mushroom; furthermore, these enzymes could be the predominant enzymes that form the dark pigments in the fruiting-body lysate. Nevertheless, the color of the fresh mushroom fruiting body did not change in spite of these highly expressed proteins. The enzyme activities of PPOs, including tyrosinases and laccases, could be strongly inhibited by the endogenous antioxidant inhibitory compounds in the mushroom metabolites. However, in chromatography, PPO enzymes with high molecular weights in the mushroom lysate were separated into low-molecular-weight inhibitors of metabolites when spontaneously exposed to oxygen; thus, these enzymes could be activated for the dark browning reaction.

The profile analysis of the mushroom metabolites in the different development phases revealed putative antioxidant compounds (Table 1) as well as enzymatic browning substrates (Table 2). Interestingly, some phenolic compounds, such as caffeic acid and chlorogenic acid, could be potential antioxidants as well as substrates of PPOs in the browning reaction in the mushroom fruiting-body extract. Adenine, a purine nucleobase, was also found to be a bilateral substrate in the mushroom. However, there were few data about how these compounds function as antioxidants or browning-reaction substances under certain conditions. Interestingly, contents of the endogenous tyrosinase inhibitors ascorbic acid and quinic acid were high only in the fruiting body (Table 1). Of these substances, ascorbic acid is already a well-known compound and additive that effectively prevents browning due to tyrosinase in *A. bisporus* [35]. Quinic acid is known to be an inducer of the antioxidant mechanism that increases urinary excretion of tryptophan and nicotinamide in the mammalian system [36], but it is also unknown whether this compound might play a similar role in mammals. Other antioxidant substances, such as ergosterol and ergothioneine, with the ability to scavenge free radicals and reactive oxygen species [37] as well as to chelate divalent metal ions [38], could inhibit the enzymatic browning reaction of PPOs in the mushroom fruiting body [39,40]. 

Indeed, these antioxidant compounds from mushrooms are already known for their significant bioactivity [19,20,41,42]. Antioxidant metabolites, mainly ascorbic acid, ergosterol, and ergothioneine, in the *H. erinaceus* fruiting body could prevent the oxidation of polyphenolic compounds and serve as anti-browning substances. Moreover, these functional compounds are potentially useful nutrient components themselves, as a food source to help the organism decrease oxidative stress [5,43]. Other potential applications of these active mushroom compounds include acting as natural ingredients for cosmetics because of their antioxidant, anti-tyrosinase, and anti-collagenase activities, among others [42,44]. Mushroom-based ingredients for skincare or anti-aging to fight against UV radiation and free radicals are already commercially available in cosmetic markets [42]. Mushroom metabolites identified as tyrosinase inhibitors, antioxidants/free-radical-scavenging compounds, and polyamines could be potential nutrient substances as well as cosmetic ingredients for nutricosmetics, combining foods, cosmetics, and pharmaceuticals [42,43,44].

## 3. Material and Methods

### 3.1. Mushroom Strains and Culture Conditions

*Hericium erinaceus* mushrooms were obtained from a local mushroom farm (Seojong, Korea). *H. erinaceus* mycelia isolated from the mushroom fruiting body were molecularly identified as NEU-2L and cultivated in potato dextrose broth (Difco, Detroid, MI, USA) at 20 °C for 20 days. The fruiting bodies and mycelia were collected, frozen immediately in liquid nitrogen, and then stored at −80 °C for total RNA extraction and preparation of the mushroom lysate.

### 3.2. Preparation of the Mushroom Fruiting-Body Lysates

The fruiting bodies were ground into a fine powder with a mortar and pestle while being cooled by liquid nitrogen. The powder was then suspended in 10 mM Tris-HCl buffer (pH 8.0), which contained 1 mM phenylmethylsulfonyl fluoride (PMSF) and a cOmplete™ EDTA-free protease inhibitor cocktail (Roche, Basel, Switzerland), overnight for extraction. The homogenate was centrifuged at 10,000×*g* for 30 min, and the resultant supernatant was centrifuged again at 20,000× *g* for 60 min. The clarified solution was used for ion-exchange chromatography.

### 3.3. Fractionation of the Mushroom Fruiting-Body Lysate Using Ion-Exchange Chromatography

The clarified crude lysate was applied to the purification columns. The crude protein solution was applied to a diethylaminoethanol (DEAE)-sepharose column (2.6 × 20 cm) (GE Healthcare, Chocago, IL, USA) equilibrated with 10 mM Tris-HCl buffer (pH 8.0) containing 1 mM PMSF and a protease-inhibitor cocktail. After the unbound proteins were washed with five column volumes of buffer, the protein fractions were eluted with three bed volumes of the buffer, followed by either a linear gradient of 0.0–1.0 M NaCl or a step gradient of 0.25 M, 0.5 M, 1.0 M, and 2.5 M NaCl. Fractions (7 mL) were collected at a flow rate of 1 mL/min. The fractions were stored at 4 °C.

### 3.4. Browning Pigment Analysis

For ultraviolet-visible (UV-vis) spectroscopy analysis, the protein purification fractions (100 μL) collected in the DEAE-sepharose column were transferred to a 96 well polystyrene microtiter plate (Greiner Bio-one, Monroe, LA, USA). The microtiter plate was scanned at room temperature by a Synergy^TM^ H1 Multi-Mode Reader (BioTek, Winooski, VT, USA) microplate reader, using wavelengths ranging from 210 to 750 nm.

For the fluorescence spectroscopy analysis, the protein purification fractions (100 μL) were transferred to a 96 well black microtiter plate (Greiner Bio-one, Monroe, LA, USA). The microtiter plate was also scanned by a Synergy^TM^ H1 Multi-Mode Reader microplate reader. For identification of the optimum excitation wavelength, the emission wavelength (Em) was fixed at 460 nm, and then excitation wavelengths (Ex) ranging from 250 to 420 nm were scanned by the microplate reader. For identification of the optimum Em wavelength, the Ex wavelength was fixed at 380 nm, and then Em wavelengths ranging from 450 to 650 nm were scanned by the microplate reader. Finally, all of the fractions with dark color pigment were measured at the optimum Ex and Em wavelength conditions (Ex_380nm_~Em_444nm_). 

### 3.5. RNA Sequencing of the Mushroom Fruiting Body

RNA sequencing with total RNA extracted from the fruiting bodies and mycelia was performed as described previously [45]. The procedure is described here briefly. Total RNA were extracted from the mushroom mycelia and fruiting bodies using the RNeasy Plant Mini Kit (Qiagen, Hilden, Germany) according to the manufacturer’s protocol, and then the extracted RNAs were further treated with DNase I. The DNase-I-treated RNA samples were purified using an RNeasy column (Qiagen, Hilden, Germany). The RNA quality and quantity were analyzed by UV spectrophotometry and gel electrophoresis. The cDNA library was constructed using a TruSeq RNA sample prep kit v2 (Illumina, San Diego, CA, USA), and RNA sequencing with an Illumine HiSeq 4000 sequencing system (Illumina, San Diego, CA, USA) was performed by MACROGEN (Seoul, Korea).

### 3.6. Tyrosinase Activity Assay

Among polyphenol oxidases, tyrosinase activity with 4-*tert*-butylphenol (Sigma-Aldrich, St. Louis, MI, USA) as a substrate was determined by measuring the amount of *t*-butyl-quinone oxidized by the enzyme. The enzyme activity was assayed in a total volume of 100 μL with 1 mM substrate in a 96 well microtiter plate at 30 °C for 30 min. After the enzymatic reaction, the oxidized monophenol product was measured on a Synergy^TM^ H1 Multi-Mode Reader microplate reader at 400 nm. One unit of tyrosinase was defined as the amount of the enzyme required to produce 1 μmol of *t*-butyl-quinone (ε_400 nm_ = 1150 M^−1^cm^−1^) per min under the reaction conditions. The background oxidation of the substrate was deduced by using a reference sample with an identical composition to the reaction mixture without the enzyme.

### 3.7. Deactivation or Inactivation of the Polyphenol Oxidases’ Activities in the Mushroom Lysate

For deactivation of the tyrosinase activities, the mushroom lysate was treated by heating at 100 °C. After heat treatment, residual enzyme activities were assayed under standard conditions after incubating the mushroom lysate for the appropriate time. 

For the inactivation of enzyme activities, the lysate was treated with a chelating agent, ethylenediaminetetraacetic acid (EDTA), at concentrations ranging from 1 to 25 mM. The appropriate concentration of EDTA was added to the lysate, and then the sample was incubated at room temperature for 1 h. After incubation, residual enzyme activities were measured using the standard assay described above. All enzyme activities were determined in triplicate. 

### 3.8. Metabolite Extracts and Analysis in the Mushroom Fruiting Body and Mycelium

Metabolome measurements were carried out by Human Metabolome Technology (HMT) Inc. (Tsuruoka, Japan). For capillary electrophoresis time-of-flight mass spectrometry (CE-TOF-MS) analysis, the frozen hydrate sample (approximately 30 mg of each sample) was mixed with 500 μL of methanol containing internal standards (50 μM) and homogenized using a homogenizer (1500 rpm, 120 s × 2 times). Subsequently, 500 μL of ultrapure water was added to the homogenates, mixed thoroughly, and centrifuged at 2300× *g* for 5 min at 4 °C. The clarified solution was filtrated through a 5 kDa cut-off filter (ULTRAFREE-MC-PLHCC, HMT Inc., Yamagata, Japan) to remove macromolecules. The filtrate was centrifugally concentrated and resuspended in Milli-Q ultrapure water for CE-TOF-MS analysis at HMT. CE-TOF-MS was carried out using an Agilent CE Capillary Electrophoresis system (Agilent Technologies, Waldbronn, Germany) equipped with an pump an Agilent 6210 time of flight mass spectrometry, an Agilent 1100 isocratic HPLC pump, an Agilent G1603A CE-MS adapter kit, and an Agilent G1607A CE-ESI-MS spray kit. System were controlled using Agilent G2201AA ChemStation software version B.03.01 for CE. Metabolites were analyzed by using a fused silica capillary (50 μm × 80 cm), with commercial electrophoresis buffer (Solution ID: H3301-1001 for cation and I3302-1023 for anion analysis; HMT. Inc.) as the electrolyte. Samples were injected at 50 mbar for 10 s for cation analysis and 22 s for anion analysis. The spectra were scanned from *m/z* 50 to 1000.

For liquid chromatography time-of-flight/mass spectrometry (LC-TOF-MS) analysis, the frozen hydrate sample was mixed with 500 μL of 1% formic acid in acetonitrile (v/v) containing internal standards (10 μM) and homogenized using a homogenizer. The mixture was homogenized once more after adding 167 μL of Milli-Q ultrapure water, and then centrifuged at 2300× *g* for 5 min at 4 °C. After the supernatant was collected, an additional 500 μL of 1% (v/v) formic acid in acetonitrile and 167 μL of ultrapure water were added to the precipitation. The homogenization and centrifugation were performed as described above to extract the residual compounds in the precipitant. The supernatant was combined with the previously collected sample. The mixed supernatant was filtrated through a 3 kDa cut-off filter to remove proteins and filtrated through a solid-phase extraction column (Hybrid SPE phospholipid 55261-U Supeleo, Bellefonte, PA, USA) to remove phospholipids. The filtrate was desiccated and resuspended in 20 μL of 50% (v/v) isopropanol in ultrapure water for LC-TOF-MS at HMT, as described above. LC-TOF-MS was carried out using an Agilent 1200 series RRLc systems SL (Agilent Technologies, Waldbronn, Germany) coupled with an Agilent-6224 TOF mass spectrometer equipped with an electrospray interface. Chromatographic analysis was performed on an Agilent Zorbax ODS column (2 mm × 50 mm, 2 μm). The autosampler and column were maintained at 4 and 40 °C, respectively. The mobile phase was water with 0.1% acetic acid (A) and water/ isopropanol/ acetonitrile (5/65/30) with 0.1% acetic acid and 2 mM ammonium acetate (B). The elution gradient was optimized as follows: 0–0.5 min, 1% B; 0.5–13.5 min, 100% B; 13.5–20 min, 100%, which was delivered at 0.3 mL min^−1^. Re-equilibration duration was 7.5 min between individual runs. The optimized conditions of TOF/MS were: capillary voltage, 4.0 kV/−3.5 kV; drying gas (N_2_) temperature, 350 °C; drying gas (N_2_) flow rate, 10.0 L min^−1^; Oct RFV, 750 V; skimmer voltage, 65 V; fragmentor voltage, 175 V; nebulizer pressure, 40 psi; scan range, *m/z* 100–1700.

For analysis of scanned metabolites, peaks detected in CE-TOF-MS and LC-TOF-MS were extracted using the automatic integration software MasterHands software version 2.17.1.11 (Keio University, Tsuruoka, Japan). Signal peaks corresponding to isotopomers, adduct ions, and other product ions of known metabolites were excluded, and the remaining peaks were annotated with putative metabolites from the HMT metabolite database on the basis of their migration times (MTs) in CE-TOF-MS or retention times (RTs) in LC-TOF-MS and *m/z* values determined by TOF-MS. In addition, peak areas were normalized against those of the internal standards, and the resultant relative area values were further normalized by the sample amount.

## 4. Conclusions

The dark browning pigment in the extract of the edible mushroom *H. erinaceus* was identified as potential byproducts of oxidization by endogenous polyphenol oxidases, including tyrosinases and laccases, measurable by fluorescence spectroscopy with 380 nm and 444 nm excitation and emission wavelengths, respectively.

Moreover, chelation deactivation and heat inactivation clearly showed that a tyrosinase—a metalloprotein as well as a PPO-family enzyme—affected the enzymatic browning reaction in the mushroom lysate. Furthermore, the identification of the putative PPO-coding genes and the analysis of their transcripts in the different growth phases suggested that the highly expressed tyrosinase and laccase isoenzymes in the mushroom fruiting body could be potential PPOs involved in the enzymatic browning reaction. The metabolite analysis of these growth statuses of the mushroom presented a number of prospective compounds as enzymatic browning substances, and they were grouped into amino acids and their derivatives, phenolic compounds, and purine and pyrimidine nucleobases. Nevertheless, the mushroom contained relatively high amounts of natural antioxidant compounds for the inhibition of tyrosinase and the scavenging of free radicals. These endogenous antioxidant substances could effectively decrease the browning reaction via PPO-inhibitory mechanisms in the mushroom fruiting body. With further evaluation of their health-promoting effects, these antioxidant metabolites and related compounds in *H. erinaceus* could be used to develop nutrient substances for healthcare and cosmetic ingredients for nutricosmetics.

## Figures and Tables

**Table 1:** Putative metabolites for the natural polyphenol oxidase inhibitors in *H. erinaceus* fruiting body and mycelium.

Compound	Relative Peak Area ^1^		*m/z* ^2^	MT/RT ^3^
	Fruiting Body	Mycelium		
Antioxidant				
Tyrosinase inhibitor				
Ascorbic acid	640	-	175.025	8.34
Caffeic acid	88	-	179.035	8.88
Chlorogenic acid	92	-	355.107	5.51
Cucumin	45	-	269.134	10.32
Pyridoxal	6	-	166.066	8.52
Quinic acid	6400	-	191.058	8.07
Terephtalic acid	110	7.4	165.020	16.38
Vanillic acid	-	4.0	167.035	8.71
Antioxidant, free radical scavenger				
Adenosine	510	560	268.105	9.60
Ergosterol	1300	1300	379.339	15.63
Ergothioneine	2900	100	230.096	17.29
Glutathione (GSH)	12	240	308.092	12.98
5-Hydroxytryptophan	20	3.7	221.094	11.20
Hypotaurine	33	-	110.027	18.06
Tartaric acid	180	-	149.011	22.05
Polyamine				
1,3-Diaminopropane	19	-	75.091	4.31
Putrescine	16	1.3	89.107	4.58
Spermidine	190	2.4	146.165	4.40

^1^ Peak area × 100,000 divided by the internal standard peak area of metabolic compounds in the two growth stages (the mushroom fruiting body and mycelium) ^2^ MS scan range: *m/z* 50~1000 ^3^ MT (migration time) in CE/RT (retention time) in LC.

**Table 2:** Potential substrates in the enzymatic browning reaction in the *H. erinaceus* fruiting body and mycelium.

Compound	Relative Peak Area		*m/z* ^2^	MT/RT ^3^
	Fruiting Body ^1^	Mycelium ^1^		
Enzymatic browning substrates				
Amino acid and its derivative				
Indole-3-carboxyaldehyde	-	170	146.062	6.91
Kynurenine	100	5.5	209.093	9.32
Phenylalanine	9400	1500	166.086	10.44
Quinolinic acid	160	-	166.016	15.47
Tryptamine	6.9	-	161.107	8.15
Tryptophan	520	110	205.097	10.69
Tyrosine	4900	280	182.081	11.09
Phenolic compounds				
*o*-Aminophenol	-	2.7	110.060	7.4
*o*-Hydroxybenzoic acid	-	2.1	137.025	10.10
*2*-Phenylethylamine	19	-	122.097	7.52
*3*-Phenylpropionic acid	-	5.6	149.062	8.60
*p*-, *m*-, *o*-Toluic acid	-	33	135.046	8.91
Purine and pyrimidine nucleobases				
Purine base				
1-Methyladenosine	4.8	3.0	282.119	9.39
2′-, 5′-Deoxyadenosine	-	2.1	252.111	9.09
3′-AMP	-	2.9	346.059	9.42
5′-Deoxy-5′-methylthioadenosine	-	2.2	298.097	9.49
AMP	290	11	346.058	9.02
ADP	260	33	426.024	10.52
ATP	270	33	505.991	11.36
Adenine	-	9.5	136.062	7.20
GMP	33	-	362.051	8.97
GDP	-	3.3	442.020	10.40
GTP	41	-	521.986	11.24
Guanosine	100	380	284.100	12.27
Hypoxanthine	23	14	137.046	10.45
IMP	-	1.8	347.038	9.28
Inosine	-	25	269.090	18.37
Xanthine	-	13	153.042	18.35
Pyrimidine base				
Cytidine	32	33	244.093	9.12
UMP	35	3.6	323.032	9.51
UDP	-	6.7	402.998	11.22
UTP	-	7.5	482.962	12.10
Uracil	-	17	113.036	20.08
Uridine	29	210	245.078	20.08

^1^ Peak area × 100,000 divided by the internal standard peak area of metabolic compounds in the two growth stages (the mushroom fruiting body and mycelium) ^2^ MS scan range: *m/z* 50~1000 ^3^ MT (migration time) in CE/RT (retention time) in LC.
